# Implementing shared decision-making in nutrition clinical practice: A theory-based approach and feasibility study

**DOI:** 10.1186/1748-5908-3-48

**Published:** 2008-11-05

**Authors:** Sophie Desroches, Marie-Pierre Gagnon, Sylvie Tapp, France Légaré

**Affiliations:** 1 Centre de recherche du Centre Hospitalier Universitaire de Québec (CHUQ), Hôpital St-François d'Assise, 45, rue Leclerc, Québec, G1L 3L5, Canada

## Abstract

**Background:**

There are a growing number of dietary treatment options to choose from for the management of many chronic diseases. Shared decision making represents a promising approach to improve the quality of the decision making process needed for dietary choices that are informed by the best evidence and value-based. However, there are no studies reporting on theory-based approaches that foster the implementation of shared decision making in health professions allied to medicine. The objectives of this study are to explore the integration of shared decision making within real nutritional consultations, and to design questionnaires to assess dieticians' intention to adopt two specific behaviors related to shared decision making using the Theory of Planned Behavior.

**Methods:**

Forty dieticians will audiotape one clinical encounter to explore the presence of shared decision making within the consultation. They will also participate to one of five to six focus groups that aim to identify the salient beliefs underlying the determinants of their intention to present evidence-based dietary treatment options to their patients, and clarify the values related to dietary choices that are important to their patients. These salient beliefs will be used to elaborate the items of two questionnaires. The internal consistency of theoretical constructs and the temporal stability of their measurement will be checked using the test-retest method by asking 35 dieticians to complete the questionnaire twice within a two-week interval.

**Discussion:**

The proposed research project will be the first study to: provide preliminary data about the adoption of shared decision making by dieticians and theirs patients; elicit dieticians' salient beliefs regarding the intention to adopt shared decision making behaviors, report on the development of a specific questionnaire; explore dieticians' views on the implementation of shared decision making; and compare their views regarding the implementation of shared decision making in different clinical settings.

It is anticipated that the results generated by the proposed research project will significantly contribute to the emergence of shared decision making in nutrition through a theory-based approach.

## Background

### Diet-based treatment options: the case of cardiovascular diseases

According to the World Health Organization, poor nutrition is among the top 10 risk factors contributing to global mortality [1]. Of the global burden of diseases attributable to poor dietary habits, about 85% was from cardiovascular diseases (CVD) and 15% from cancers. One way through which dietary treatment effectively manages CVD is by reducing high plasma cholesterol levels [2], the primary modifiable risk factor for CVD [3]. While earlier dietary recommendations provided by the American Heart Association focused primarily on lowering total fat and cholesterol dietary intake [4], their most recent versions advocate alternatives to the traditional low-fat diet [5,6] that have been referred to as 'inclusive food-based approaches' [7]. In that regard, recent studies have shown the remarkable cholesterol-lowering properties of the Portfolio diet [8]. Consumption of a traditional Mediterranean-style diet has also shown favorable effects on lipoprotein levels, as well as myocardial and cardiovascular mortality [9]. As therapeutic eating plans have traditionally been considered restrictive, the increasing number of dietary options represents an incredible opportunity to better individualize dietary treatment in order to match patients' preferences, values, lifestyle, and global health status. On the other hand, the rapid and unregulated amount of nutrition messages that is currently emerging in cyberspace, television, newspapers and magazines is confusing for the general population [10]. It is in this context of increased number of dietary options that the need for patient guidance from dieticians is expected to increase in the near future.

### Current state of knowledge about the integration of shared decision making key elements in dieticians' clinical practice

Shared decision making (SDM) is defined as a decision making process jointly shared by patients and their health care providers [11]. It aims at helping patients play an active role in decisions concerning their health, which is the ultimate goal of patient-centered care [12]. SDM includes the following components: taking into account the establishment of a context in which the values and preferences of the patient are sought and deemed necessary, reviewing the patient's preferences for role in decision making, and the existence and nature of any uncertainty about the course of action to take [13]. SDM helps patients base their preference on the best evidence of the risks and benefits of all the available options, and makes explicit the component of uncertainty in the clinical decision-making process [14].

Interestingly, recent evidence supports an emerging interest in the key elements of SDM in the dietetics literature. One of the crucial components of SDM is that it rests on the best evidence of the risks and benefits of all the available options [13]. The first scientific data referring to evidence-based practice (EBP) within the dietetic profession were published in 1998 [15,16]. In spite of this recent interest, both the American Dietetic Association and the Dieticians of Canada have been actively promoting the development of EBP in dieticians' practice through online tools featuring summaries of the best available research on nutrition, as well as several EBP nutrition guidelines [17,18]. Despite these efforts, a recent study indicated that although dieticians recognized the value of research for practice, they lacked both time and ability to critically read research [19].

Informed and SDM have been referred to as 'the crux of patient-centered care' [20]. Following the example of EBP, patient-centered care within dieticians practice is fairly new and apparently not fully understood nor integrated into day to day practice [21]. Nevertheless, it is included in the current Professional Standards for Dieticians in Canada,[22] in which it is defined as 'the use of collaborative and partnership approaches where the client's own experience and knowledge are central and carry authority within the client-professional partnership'. A recent study reported that dieticians perceived some barriers to the implementation of a patient-centered approach, including their own struggle to recognize patients' expertise and the lack of applicability due to their workplace characteristics [21].

Although the abovementioned studies provide some insight at the perception of dieticians regarding EBP and patient-centered care, they do not explicitly refer to SDM. Most importantly, they are lacking the theory-based knowledge necessary to unravel the underlying factors that should be targeted to develop effective interventions aiming at implementing SDM in dieticians' clinical practice. Indeed, a search through the 5,600 clinical trials registered by the Cochrane Effective Practice and Organisation for Care Group (EPOC) revealed that only one study aimed to objectively measure dieticians' practice within a real videotaped clinical setting [23], thus urging the need to conduct studies targeting dieticians' clinical practice, and most specifically studies aiming at the implementation of SDM in dieticians clinical practice.

### Relevance of investigating the implementation of shared decision making into allied health professionals' clinical practice

In a recent study that aimed to implement SDM in primary care, Légaré *et al. *identified the nature of the decisions that were discussed in 903 clinical encounters in primary care [24]. They reported that lifestyle issues ranked as the fourth most often cited type of decisions, accounting for 5.9% of all the decisions encountered [24], thus suggesting that decisions related to lifestyle issues in primary care, which include diet-related decisions, represent a highly relevant research area for investigating SDM. However, in a systematic review by our team that identified 28 unique studies reporting on barriers and facilitators to implementing SDM in clinical practice as perceived by health professionals, the vast majority of participants (n = 2784) were physicians (89%) suggesting that there was a lack of interprofessional perspective to SDM [25]. Towle and Godolphin have proposed a framework of eight competencies for the practice of informed SDM by physicians and seven competencies for patients for informed SDM [13]. Although they were initially developed for physician/patient encounters, they hypothesized that they could apply to other health professionals. Indeed, a recently published review article presents convincing arguments pertaining to the application of this framework to physical therapists, and concludes that physical therapists could use the SDM framework to incorporate client-centred care, informed choice, and evidence-based practice [26].

### Theory-based approaches to the implementation of shared decision making in clinical settings

Social cognitive theoretical models have been used to improve our understanding of health-related behaviors, including those of professionals [27-29]. Although most SDM models referred to a set of competencies and behaviors [13], very few studies have used a theory-based approach to predict the determinants underlying a SDM-related specific behavior [30,31], and none have simultaneously investigated the operationalization of two key SDM behaviors. To the best of our knowledge, the first study using the Theory of Planned Behavior (TPB) to identify the determinants underlying the intention of physicians to screen for decisional conflict, one of the SDM-related specific behaviors, was recently published by our team. It concluded that modifiable determinants of this intention change over time, suggesting that effective implementation interventions in this area will need to be modified longitudinally [30]. Moreover, based on our ongoing international collaborative efforts toward implementation of SDM in clinical practice, we have identified an important knowledge gap regarding the assessment of SDM-related specific behaviors [32].

### Gaps in knowledge to be addressed by the proposed research

In summary, 1) a careful search through the 5,600 clinical trials registered by the Cochrane EPOC group revealed that dieticians' practices have exceptionally been assessed in real clinical settings; 2) there is an increasing number of dietary treatment options for the management of chronic diseases; 3) nutrition health professionals organizations in Canada and the United States have clearly acknowledge the importance of EBP and patients views, two key elements of SDM, in dieticians' clinical practice; 4) few studies have investigated the integration of SDM-related specific behaviors in health professionals allied to medicine, thus potentially hampering the implementation of SDM in various healthcare contexts [25]; and 5) theory-based approaches to operationalize SDM-related specific behaviors that are the most influential for improving decision quality have yet to be thoroughly investigated [33].

## Design and methods

### Design

A three-phase mixed methods study with a transversal design will be conducted.

### Participants

For phases one and two (see below), dieticians having inpatient or outpatient clinical activities will be recruited at the three sites of the Quebec University Hospital Center (N = 40). The inclusion criterion for participating in this study will be to be a member of the Professional Order of Dieticians of Quebec, the province of Quebec's professional regulatory body. For phase three, a sample of 35 dieticians will be recruited among a total of 2,359 members from the Professional Order of Dieticians of Quebec. All dieticians recruited will be French-speaking.

### Data collection procedures

#### Phase one: Quantitative component study

At study entry, all participating dieticians will complete a questionnaire assessing their social and professional characteristics, as well as their preferred role in decision making [34]. They will be asked to audiotape at least one clinical encounter in which they think a value-sensitive dietary treatment-related decision will take place. Consent will be provided by both dietician and patient. The OPTION (Observing patient involvement) scale (Cronbach's alpha = 0.79) [35] will be used in order to assess the integration of SDM key elements within clinical encounters. This third-observer 12-item scale assesses to what degree clinicians involve patients in decision making. [36]. Following consultations, dieticians will be invited to complete a questionnaire that will provide information on the decision that was made, the decisional conflict as measured with the Decisional Conflict Scale (DCS; (Cronbach's alpha = 0.78–0.90)) [37], and their perception of the decision's quality. The patients' questionnaire will assess their preferred role in decision making [34], the decision that was made, their decisional conflict (DCS; Cronbach's alpha = 0.78–0.92) [38,39], their perception of the decision's quality, and sociodemographic data.

#### Phase two: Qualitative component study

Dieticians will then participate in a 90-minute interactive workshop (five to six groups, n = 6 to 7 participants/group for a maximum of 40). A 30-minute didactic presentation will first introduce the relevance and basic concepts of SDM. Then, a 60-minute focus group will be conducted and structured around three main issues.

**Figure 1:**
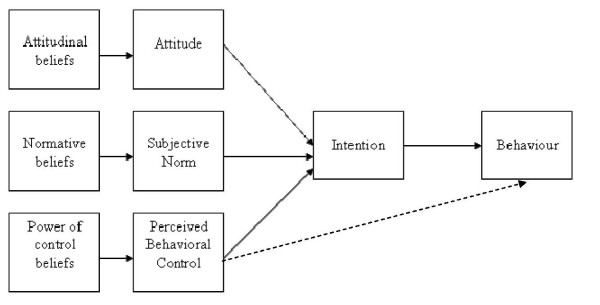
**TPB model**. The TPB was proposed by Icek Ajzen as an extension of the theory of reasoned action. It is applied to study the relations among beliefs, attitudes, behavioral intentions, and behaviors. According to this theory, human behavior is the result of three different beliefs: behavioral (beliefs about the likely consequences of behavior), normative (beliefs about expectations of others), and control (beliefs about the factors that may facilitate or impede the adoption of the behavior). These beliefs are determinants of the attitude toward the behavior, the subjective norm, and perceived control, which are the factors that predict the intention of performing a given behavior. In our research, based on TPB, we will identify salient beliefs pertaining to the adoption of two SDM-related specific behaviors in nutritional clinical practice.

In line with the TPB (Figure 1), the first issue will seek to elicit dieticians' salient beliefs regarding:

1. Attitude: the advantages and disadvantages to present the evidence-based dietary treatment options in the context of clinical encounters with patients.

2. Subjective norm: the individuals or groups of individuals important to them who would approve or disapprove that they present the evidence-based dietary treatment options in the context of clinical encounters with patients.

3. Perception of behavioral control: barriers or facilitators associated with presenting the evidence-based dietary treatment options in the context of clinical encounters with patients.

Also, in a similar fashion, dieticians will be invited to discuss their salient beliefs pertaining to a second behavior,*i.e.*, in the context of clinical encounters with patients, to help patients clarify their values, or what is most important for them regarding the advantages and disadvantages of the evidence-based dietary treatment options.

In response to the didactic presentation, dieticians will be asked about their views regarding the implementation of SDM in their own clinical practice, including the obstacles, from their perspectives, that face their patients when making nutrition-related decisions [40].

#### Phase three: Elaboration and testing of the questionnaires

In line with the TPB [41], the most frequent salient beliefs underlying the determinants of the intention to perform each of the two targeted SDM-related specific behaviors will be used to elaborate the items of the questionnaires. Therefore, attitude, perceived subjective norm, and perceived behavioral control (Aact, SN, PBC) will be first individually assessed on a varying number of belief-based variables. Then, the behavioral intentions of interest and their determinants (Aact, SN, PBC) will also be assessed by means of three questions, each using a five point scale ranging form -2 (*e.g.*, very likely) to +2 (*e.g.*, very likely). A mean composite score will be computed.

### Sample size and analysis plan

For phase one, 40 dieticians will help us identify the feasibility of a larger study as well as the parameters for calculating a proper sample size (mean OPTION score and mean DCS score ± their respective SD). Decisional conflict score of dieticians and patients will be compared and levels of agreement of the patient-dietician dyads on the DSC will be measured [24]. OPTION scores will be correlated with decisional conflict score of dieticians and patients, and with their agreement score. Structural Equation Modelling (SEM) will be performed to assess how subscales from the DCS relate to each other [42]. Also, OPTION scores from dieticians having inpatient clinical activities and those from dieticians having outpatient activities will then be compared in order to assess whether they differ according to the inpatient or outpatient character of the nutrition consultation.

For phase two, it is estimated that approximately 40 dieticians will be needed to identify the salient beliefs in order to reach saturation. All focus groups will be audiotaped and transcribed verbatim. Content analysis of focus groups will be performed independently by two individuals. In line with the first and second issues discussed during the focus groups interviews, data will be analyzed with the purpose of eliciting the indirect (belief-based) measures for all the TPB (Figure 1) constructs (Aact, SN, PBC). Both assessors will read the material that will be analyzed into themes (attitudinal beliefs, normative beliefs, and control beliefs). Each set of beliefs will be converted into sets of statements. In line with the third issue discussed in the focus groups, views pertaining to the implementation of SDM in dieticians' clinical practice will be coded according to a pre-established list of codes based on a taxonomy of barriers to the implementation of clinical practice guidelines [43], and on a list of barriers and facilitators generated by a study from our research group [44].

For phase three, questionnaires will be first pilot-tested by five dieticians for comprehension and clarity (face validity). A test-retest reliability study will be performed with a new group of 35 dieticians. Internal consistency (Cronbach's alpha) will be assessed. Reliability will be assessed with the intra-class correlation coefficient. Questionnaires will first be developed in French, but we plan to eventually engage in back translation activities based on the guidelines for cross-cultural adaptation of self-reported measures [45], and our previous expertise in this type of research activity [46,47]. Descriptive analyses of participants will be performed with SAS (SAS Institute Inc., Cary, N.C., USA). SEM will be performed to assess how both specific behaviors and their respective determinants relate to each others.

### Ethical considerations

Ethics approval for the project was received from the Research Ethics Board of the Centre Hospitalier Universitaire de Québec (approved 24 July 2008; ethics number 5-08-06-02).

## Discussion

In line with the specific objectives and observed gaps in knowledge, the proposed research project will be the first study to: 1) provide preliminary data to design and conduct a larger trial to evaluate the adoption of SDM by dieticians and theirs patients in the context of diverse nutritional clinical practice situations; 2) elicit dieticians' salient beliefs regarding the intention to adopt two behaviors related to SDM; 3) report on the development of two questionnaires, which in turn will eventually be administered to a larger representative sample of dieticians to identify and assess the relative importance of the determinants that predict the intention to perform two behaviors necessary for SDM; 4) explore dieticians' views on the implementation of SDM that will help develop effective interventions to foster SDM in nutrition clinical practice; and 5) compare the views of dieticians regarding the implementation of SDM in two different clinical settings – inpatient and outpatient – that will help develop interventions tailored to fit a given nutritional clinical context. It is anticipated that the results generated by the proposed research project will significantly contribute to the emergence of SDM in nutrition through a theory-based approach. The reform of primary health care in Canada and abroad presents new opportunities for dieticians to contribute to disease prevention and management in interdisciplinary and collaborative primary health care settings[48]. The implementation of SDM within educational and clinical environments of multiple health professions allied to medicine, including dieticians, will be key to promoting an integrated approach to primary health care decision-making.

## Competing interests

The authors declare that they have no competing interests.

## Authors' contributions

SD conceived and developed the study, drafted the study protocol, and leads the study under the supervision of FL. FL and MPG helped to draft both the study protocol and this manuscript. ST developed the questionnaires, prepared the ethical approval document, coordinates the study and helped to draft the manuscript. SD and ST are responsible for the data collection. All authors read, and approved the final manuscript. FL and SD are its guarantors.
